# Aluminum

**Published:** 1867-05

**Authors:** James B. Bean


### ARTICLE VII.

### *Aluminum.*

By James B. Bean, D.D.S.

This metal is named from *alumen*, the Latin term for alum, which is a double salt of potassa and the earth alumina combined with sulphuric acid. The name *aluminium* has been adopted by some writers, but we can see no just reason for this orthography; *alumium* would have been more consistent with our own word *alum;* and for the sake of euphony or uniformity of termination, we might also say *platinium* or *tantalium* in place of platinum or tantalum.

The chemical symbol of aluminum is Al., its combining number is 13.7 and its specific gravity about 2.30 when cast, but may be increased to 2.60 by hammering or rolling.

The sesquioxide, or alumina is the only compound of this metal with oxygen, and is so called because of its isomorphism with the sesquioxide of iron. The metal aluminum thus combined with oxygen and other elements is more abundant on the earth than any other of this class of bodies that has been rendered useful in the arts in its metallic state. As well as being the principal component of all clays, which are combinations of sesquioxide of aluminum with silicic acid, it is a large constituent of all granites and felspathic rocks, and appears in a state of purity in the precious gems sapphire and ruby. Corundum and emery are also nearly pure alumina.

Aluminum, formerly known to chemists only as a gray powder resembling spongy platinum, is now prepared in large quantities in France and England by the decomposition of the chloride or fluoride by sodium. The chloride of aluminum is prepared on the large scale by passing chlorine over a previously ignited mixture of clay and coal-tar in retorts like those used in the preparation of coal gas, and is either made to pass into a chamber lined with plates of earthenware, where it condenses into a compact crystalline mass ; or the vapor is made to pass over chloride of sodium at a red heat, whereby it is converted into the double chloride of aluminum and sodium. To effect the reduction, 400 parts of this double salt, 200 parts of chloride of sodium, 200 parts of fluor spar, (or better cryolite,) all perfectly dry and finely powdered, are mixed together, and the mixture placed, together with 75 or 80 parts of sodium, in an earthen crucible, the saline mixture and the sodium being deposited in alternate layers. The crucible is then moderately heated till the action begins, afterwards to redness, the melted mass stirred with an earthenware rod and afterwards poured out. Twenty parts of aluminum are thus obtained in a compact lump, and about five parts in globules encrusted with a gray mass. (*H. Ste-Claire Deville.*)

Since the great improvement of Deville in the production of this metal on the large scale, it has attracted considerable attention from the manufacturing as well as the scientific world ; and within the past few years, the dental profession have looked forward with much interest to the time when aluminum should be successfully adapted to use as a base for artificial teeth. Then to a cursory view of the properties of the metal under consideration which more immediately concern its manipulation in the dental laboratory, together with the progress of the recent experiments of the writer in casting plates for artificial teeth, will conclude this brief article.

Aluminum is peculiarly suited to the construction of bases of artificial teeth, and more particularly for upper sets. When cast, in its pure state, it is less than one-fourth the weight of an equal bulk of silver, about one-eighth the weight of gold, and little more than one-tenth the weight of an equal bulk of platinum. It is nearly as strong and rigid as an equal thickness of wrought iron; it is as tenacious as ordinary cast brass, and will bend back and forth several times before breaking. Its whiteness and lustre when polished rivals that of silver, and when burnished attains a brilliancy more lasting under wear than that of silver. When heated, aluminum attains a pasty condition as it approaches a red heat, and at a cherry red it is quite fluid, yet remains very sluggish in its movements, owing to its extreme lightness and the thin skin of oxide that seems to envelope the mass. At a bright red heat it still remains unaltered by contact with the air, but at a white heat it takes fire and burns with a brilliant flame, leaving behind a mass of fused corundum. Nitric and sulphuric acids have no perceptible action on aluminum at common temperatures, but boiling nitric acid attacks it with about the rapidity that aqua regia does pure gold. Hydrochloric acid attacks it at ordinary temperatures, slowly dissolving it with the evolution of hydrogen gas. Acetic acid in a concentrated state, exposed to the open air, dissolves it very slowly after one or two day's immersion, but common vinegar has no perceptible action. Sulphuretted hydrogen, which is the most common agent that attacks silver and even gold plates in the mouth, has no action whatever on pure aluminum. Its permanence in the mouth will undoubtedly be equal to gold or platinum ; pieces that have been worn in the mouth for weeks even without cleaning, under the immediate observation of the writer, showed no action whatever by the fluids of the mouth.

Many gentlemen in the dental profession have been carefully experimenting, in view of making a successful application of this excellent metal to our purposes; but as yet, so far as is known to the writer, no one has succeeded in *casting* a satisfactory plate with teeth attached, which would fit the mouth, until his experiments proved successful a few weeks ago, after months of discouraging and laborious investigation and experimenting. The writer succeeded in casting a plate in 1860, but was unable to produce a plate with uncracked teeth attached until last November, and the prodigious shrinkage of the metal was not overcome so as to make a perfect fit to the mouth, until within the last two or three months. It is now confidently asserted, that in a very short time the process will be developed in its details, and the apparatus prepared for using it so as to offer it to the profession for successful application. Since the production of the article published in the April number of the DENTAL COSMOS, page 470, current volume, the writer has made many important improvements, and others are yet under trial, he must therefore ask his brethren of the profession to be patient, and they shall have the benefit of all of these improvements in due time. No great improvement in an art like this is quickly developed, and it is not desirable that these experiments should be given out until perfected, or at least reduced to a practical form.

For the information of dentists, the writer would state that as soon as there is a demand for the metal, it will be imported and for sale in bars at the Dental Depots. Its present price is $2.00 to $2.50 per oz., in gold; and plates will rarely weigh over one ounce. The apparatus and outfit for making aluminum work will be less expensive than that for vulcanite work, and much easier kept in order. The labor of making a set on aluminum will be about that of first-class gold work, and it gives room for an almost unlimited amount of skill in accomplishing its perfection, consequently it will largely tend to the elevation and highest developement of our art.

The writer would not use aluminum for lower sets, although it is as easily applied to these, yet its lightness and strength is its great recommendation for upper sets; and there is nothing better than pure tin, or an alloy of tin and silver, for lower sets, as we want these as compact and heavy as possible.

